# Value of Left Atrial Strain in Predicting Recurrence after Atrial Fibrillation Ablation

**DOI:** 10.3390/jcm12124034

**Published:** 2023-06-13

**Authors:** Marek Kiliszek, Beata Uziębło-Życzkowska, Krystian Krzyżanowski, Agnieszka Jurek, Robert Wierzbowski, Magdalena Smalc-Stasiak, Paweł Krzesiński

**Affiliations:** Department of Cardiology and Internal Diseases, Military Institute of Medicine—National Research Institute, 04-141 Warsaw, Poland; buzieblo-zyczkowska@wim.mil.pl (B.U.-Ż.); kkrzyzanowski@wim.mil.pl (K.K.); ajurek@wim.mil.pl (A.J.); rwierzbowski@wim.mil.pl (R.W.); msmalc-stasiak1@wim.mil.pl (M.S.-S.); pkrzesinski@wim.mil.pl (P.K.)

**Keywords:** atrial fibrillation, pulmonary vein isolation, left atrial strain, left atrial appendage velocity

## Abstract

This study tested the relationship between left atrial (LA) function parameters and the results of pulmonary vein isolation (PVI) in patients with atrial fibrillation (AF). Consecutive patients undergoing PVI for the first time between 2019 and 2021 were included. Patients underwent radiofrequency ablation using contact force catheters and an electroanatomical system. Follow-up consisted of ambulatory visits/televisits and 7-day Holter monitoring (at 6 and 12 months after ablation). On the day of ablation, all patients underwent transesophageal and transthoracic echocardiography with LA strain analysis. The primary endpoint was atrial tachyarrhythmia recurrence during the follow-up period. Of 221 patients, 22 did not meet the echocardiographic quality criteria, leaving 199 patients. The median follow-up period was 12 months, and 12 patients were lost to follow-up. Recurrences were observed in 67 patients (35.8%) after a mean of 1.06 procedures per patient. The patients were divided into a sinus rhythm (SR, *n* = 109) group and an AF (*n* = 90) group based on their cardiac rhythm at the time of echocardiography. In the SR group, univariable analysis showed that LA reservoir strain, LA appendage emptying velocity (LAAV), and LA volume index predicted AF recurrence, with only LAAV being significant in the multivariable analysis. In AF patients, univariable analysis revealed no LA strain parameters predicting AF recurrence.

## 1. Introduction

Atrial fibrillation (AF) is the most common sustained supraventricular arrhythmia. AF can cause varying degrees of symptoms, from mild to severe. Two baseline strategies are applied in terms of better symptom control: rate control and rhythm control [1]. In highly symptomatic patients, the rhythm control strategy is preferred. Catheter AF ablation is an important element of a rhythm control strategy. AF ablation is indicated in patients with symptomatic AF when (a) antiarrhythmic drugs are ineffective or intolerant, (b) as a first-line rhythm control therapy, or (c) when tachycardia-induced cardiomyopathy is suspected. The key procedure in atrial fibrillation (AF) ablation is pulmonary vein isolation (PVI) [1]. Despite significant technological progress, a substantial number of patients (20–30%) experience recurrence [2,3]. Significantly enlarged left atrial (LA) volume, advanced age, long AF duration, renal dysfunction, and other cardiovascular risk factors are considered major risk factors of AF recurrence in persistent AF [1]. Identifying patients with a high risk of recurrence could influence the extent of AF ablation or even the qualification for ablation. Since the left atrium is a key structure in AF, there is an important clinical need for a reproducible, easily accessible, and non-invasive means of quantitative analysis of the left atrium of AF patients prior to the ablation procedure. Speckle-tracking echocardiography provides detailed insights into left atrial (LA) function, making it a particularly promising method for studying these patients. Left atrial strain (LAS) analysis is an echocardiographic method based on LA myocardial deformation, which can accurately analyze all three phases (reservoir, conduit, and contraction) of LA function [4]. According to experts, this echocardiographic method can be used as a surrogate for LA dysfunction and structural fibrous remodeling [5]. Thus, the aim of this study was to assess the predictive value of LA strain measurements in AF patients undergoing PVI.

## 2. Materials and Methods

Consecutive AF patients undergoing their first PVI between 2019 and 2021 were included in the analysis. Patients qualified for AF ablation according to the following guidelines: only symptomatic AF patients were considered, with the majority of patients experiencing drug-resistant AF [1]. The patients were informed about the ablation procedure and the expected efficacy and risks, and a shared decision was made in every case.

### 2.1. Ethical Approval

The study was conducted according to good clinical practice guidelines and the Declaration of Helsinki. The study protocol was approved by the Bioethics Committee of the Military Institute of Medicine (50/WIM/2019), and each patient provided written informed consent.

### 2.2. Echocardiographic Measurements

**Figure 1:**
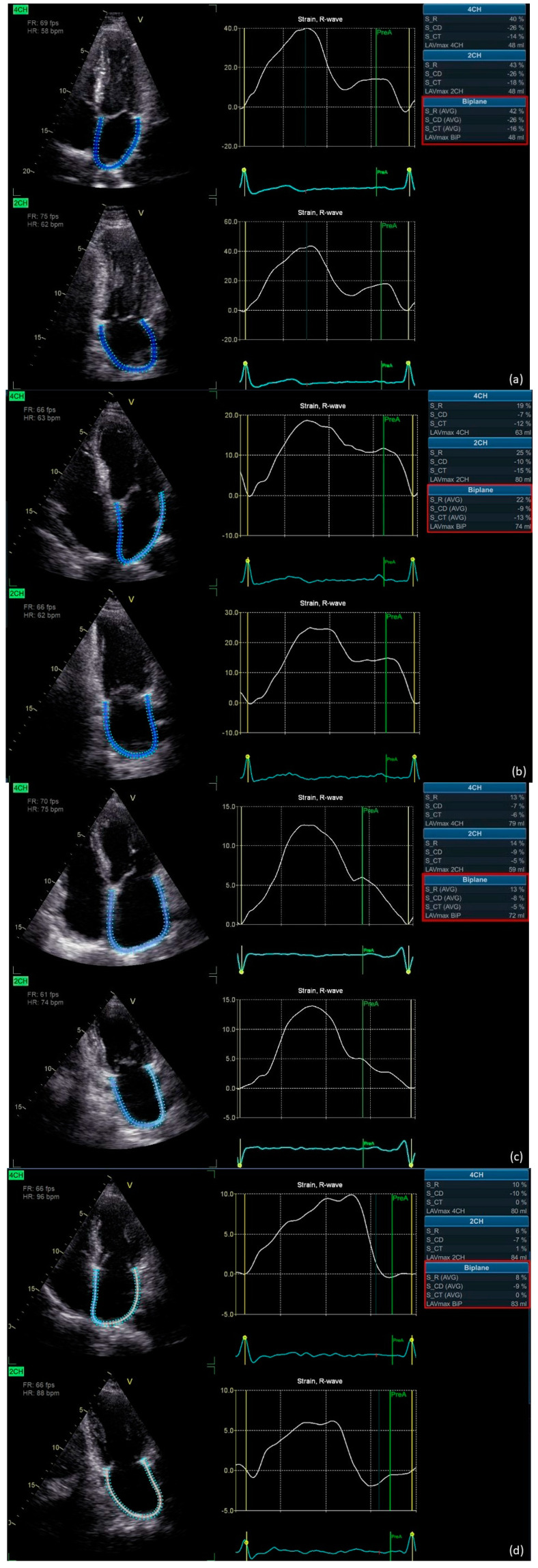
Examples of left atrial strain (LAS) values: (**a**) normal LAS in a patient with sinus rhythm during echocardiography, (**b**) slightly decreased LAS in a patient with sinus rhythm during echocardiography, (**c**) significantly decreased LAS in a patient with sinus rhythm during echocardiography, and (**d**) LAS in a patient with atrial fibrillation during echocardiography. Abbreviations: LAVmax 2CH, maximum left atrial volume in two-chamber apical view; LAVmax 4CH, maximum left atrial volume in four-chamber apical view; LAVmax BIP, maximum biplane left atrial volume; S_CD, left atrial conduit strain; S_CT, left atrial contraction strain; S_R, left atrial reservoir strain.

On the day of AF ablation, all the patients underwent transthoracic echocardiography (TTE) and transesophageal echocardiography (TEE) before the procedure. All echocardiographic examinations were performed using a high-quality echocardiograph (Vivid E95, General Electric Medical Systems, Milwaukee, WI, USA) with a 1.5–4.5 MHz transducer for TTE and a 3.0–8.0 MHz transducer for TEE. All echocardiographic measurements were obtained (using GE EchoPAC BT12) by a single experienced echocardiographer accredited by the Section of Echocardiography of the Polish Cardiac Society. The left ventricular (LV) and LA conventional echocardiographic parameters and parameters of LA deformation were measured as recommended by the contemporary guidelines [6,7]. The values of the LV strain parameters were obtained from three non-foreshortened apical chamber views (two-chamber apical view, 2AC; three-chamber apical view, 3AC; and four-chamber apical view, 4AC), and those of the LA strain parameters were obtained from two views (4AC and 2AC), ensuring a frame rate of at least 60 frames per second. The QRS wave onset was set as a reference point for assessing all LAS values (LASr, LAScd, and LASct). Left atrial strain analysis was performed using automated software specifically dedicated to LA assessment (automated functional imaging of the left atrium [AFI LA]), and LAEF was determined automatically using AFI LA. The total atrial conduction time (PA-TDI) was defined as the interval between the initiation of the P-wave on the surface electrocardiogram and the peak of the A-wave on tissue Doppler imaging of the lateral wall of the LA just over the mitral annulus. Examples of strain measurements are shown in Figure 1.

During TEE, LAAV was measured via pulsed-wave Doppler positioned 1 cm below the LA appendage orifice as the average of three consecutive beats during the AF rhythm. To assess the intraobserver variability of the LAS measurements, 20 patients were randomly selected. The intraobserver variability coefficients were calculated using images independently recoded at two different points in time by the same observer. The intraclass correlation coefficients for the intraobserver variability of LASs were 0.99 for LASr A4C, 0.98 for LASr A2C, 0.99 for LASct A4C, and 0.98 for LASct A2C. The mean difference divided by the mean of two measurements for intraobserver variability was 0.4% (−1.1 –2.0%) for LASr A4C, 0.6% (−1.6–2.8%) for LASr A2C, 0.2% (−3.7–6.6%) for LASct A4C, and 0.5% (−3.1–4.1%) for LASct A2C.

### 2.3. AF Ablation Procedure

The left atrium was accessed through a double transseptal puncture. A circumferential mapping catheter and an irrigated contact force catheter were used for mapping and radiofrequency (RF) ablation. Navigation of the catheters was based on fluoroscopy and an electroanatomical system (CARTO 3, Biosense Webster, Irwindale, CA, USA, or NaviX, St Jude Medical, St. Paul, MN, USA). The ipsilateral veins were jointly isolated. The power limit was 40 W, and the respective indexes of lesion size were applied (ablation index and lesion size index). The maximal interlesion distance was 6 mm. The endpoint of the procedure was the isolation of all pulmonary veins. Ablations beyond PVI were performed only when the patient developed atrial tachycardia or atrial flutter during the procedure. After the isolation of all veins, the veins were rechecked after a waiting period of 15–20 min.

### 2.4. Follow-Up

Recurrence was defined as any atrial tachyarrhythmia lasting more than 30 s [8]. A three-month blanking period was applied. All antiarrhythmic drugs were discontinued after catheter ablation. The patients were scheduled for follow-up visits at 6 and 12 months and yearly thereafter. All asymptomatic patients underwent 7-day Holter monitoring as part of each follow up visit.

### 2.5. Statistical Analysis

A Shapiro–Wilk test was used to test the normality of the distribution of the continuous variables. The continuous variables are presented as the median (interquartile range [IQR]) or mean (standard deviation [SD]), depending on the variable distribution. The categorical variables are presented as frequencies. The continuous variables were compared between groups using Student’s t-test or the Mann–Whitney U-test, depending on the normality of the distribution. Chi-square and Fisher’s exact tests were used to calculate differences in the categorical variables. To identify the predictors of the results of AF ablation, given that the patients had different follow-up periods, uni- and multivariable Cox regression analyses were performed. All parameters with p-values below 0.05 in the univariable analysis were included in the multivariable analysis. If highly correlated parameters were present, only one representative was chosen for the multivariable analysis, based on its p-value in the univariable analysis and its biological validity. To determine the threshold value, receiver operating characteristic (ROC) curves and AUCs were calculated for the parameters found to be significant by multivariable Cox regression analysis. Two-tailed tests were used for all calculations, and a p-value below 0.05 was considered statistically significant. Statistical analysis was performed using Statistica v. 12 (Statsoft Inc., Tulsa, OK, USA).

## 3. Results

A total of 221 patients were included in the analysis. In 22 patients, analysis was not possible due to poor visualization during echocardiography. Finally, the data of 199 patients were analyzed: 109 patients with AF who had undergone an echocardiographic study when they had a normal sinus rhythm (SR) and 90 AF patients who had undergone an echocardiographic study while experiencing AF. The characteristics of the study groups are shown in Table 1. The patients tested during AF were significantly older than those in the SR group and experienced non-paroxysmal AF more frequently. All measurable strains were distinctly different between the two groups, as were LA appendage emptying velocity (LAAV) and LA volume index (LAVI). Median follow-up was at 12 months (IQR 8.5–15.5), and 12 patients were lost to follow-up. Recurrences were observed in 67 patients (35.8%), with a mean of 1.06 procedures per patient. The vast majority of recurrences were AF; only three patients (4.5%) had atrial tachycardia. At the end of the follow-up period, 10 patients without AF recurrence (7.6%) were receiving antiarrhythmic drugs.

Due to essential differences in the echocardiographic parameters of patients tested during normal SR and those tested during AF (for LASr, 95% CI of the SR and AF groups did not overlap; see Table 1), analyses were performed separately for SR and AF patients.

### 3.1. Sinus Rhythm Patients

In a univariable Cox regression analysis, LASr and LAScd were significantly linked with AF recurrence (Table 2). Among other echocardiographic parameters, LAVI and LAAV correlated significantly with AF recurrence. Body mass index and LA pressure were also found to be significant predictors of AF recurrence. In a multivariable Cox regression analysis, only LAAV was found to be a statistically significant predictor of AF recurrence. The threshold value was 44 cm/s for LAAV and 21% for LASr (Supplementary Figures S1 and S2, area under the curve [AUC] 0.69 and 0.62, respectively).

### 3.2. Atrial Fibrillation Patients

In a univariable Cox regression analysis, none of the LAS parameters correlated significantly with AF recurrence (Table 3). LAVI, LAAV, a history of non-paroxysmal AF, and LA pressure were found to have a significant impact on AF recurrence. A multivariable Cox regression analysis revealed LAVI to be a statistically significant predictor of AF recurrence.

## 4. Discussion

The goal of this study was to assess the importance of LASs in predicting AF recurrence after PVI. In patients with normal SR during echocardiography, LASr was only moderately predictive of AF recurrence, with multivariable analysis finding LAAV to be the only independent predictor. In the patients who had undergone echocardiography during AF, none of the LASs were found to be of value in predicting AF recurrence after PVI.

It is well known that the most reliable measurements of LASs are made during normal SR [9]. However, in clinical practice, stable SR is unachievable in a substantial number of patients, and many patients with persistent AF have recurrences within days after cardioversion. Even patients with paroxysmal AF may have numerous AF episodes that are difficult to control. As a result, some studies compared the echocardiography data of AF patients experiencing normal SR during testing with those experiencing AF [10,11].

However, the joint analysis of SR and AF patients has drawbacks. For example, a reliable threshold point for differentiating patients with low and high risk cannot be found due to obvious differences in the values of LAAV and LASr during AF and SR, with many parameters unable to be calculated during AF at all. In a previous study, we established that, due to the large differences in LASr during SR and AF, such patients should be studied separately [12]. In addition, most patients that must be tested during AF, have long-standing persistent AF, significantly decreased LASr compared to SR patients, and a higher risk of recurrence after ablation, which could bias the results. Similar problems may occur in terms of LAAV. Therefore, for the current study, we analyzed patients experiencing AF and SR during echocardiographic testing separately, regardless of the underlying type of AF.

In the SR group, LASr, LAScd, LAVI, and LAAV were univariable predictors of AF recurrence after catheter ablation. Multivariable analysis revealed only LAAV to be significant. This finding is supported by numerous publications showing the importance of LASs in predicting the results of AF ablation. For example, peak atrial longitudinal strain was revealed as a predictor of AF recurrence after cryoballoon ablation [8], and in a meta-analyses [13,14]. LASr and LA contraction strain (LASct) predicted AF recurrences in a large cohort of patients undergoing AF ablation [15]. Another study found the risk of AF recurrence after catheter ablation to be significantly higher in patients with low LAEF and low LASr [9]. The threshold of LASr to predict recurrence after AF ablation calculated in the current study was 21% (with moderate AUC); this was less than the 25.2% level found by Nielsen [15] and less than the lower limit of normality (23.0%) found in the Copenhagen City Heart Study [16]. It should be noted that the median value of LASr in the Copenhagen City Heart Study for the healthy population was 39%.

Interestingly, neither LASct nor LAEF were predictive of AF recurrence after AF ablation, which was in contrast to the results of Nielsen et al. [15]. Since our results for LASct and the LAEF were close to being statistically significant (with p-values between 0.051 and 0.07, respectively), the relatively small group size could be an explanation. Still, LASr seems to be a better predictor than LASct or LAEF. The predictive power of LASr in multivariable analysis with LAAV needs further clarification from larger studies. In many studies in which LASr was tested, LAAV was not measured [9,15].

The most potent predictor of AF recurrence in patients with SR is LAAV. The predictive power of LAAV after catheter ablation was shown in many populations [17,18,19,20]. This study confirmed LAAV to be a predictor for both the SR group (through uni- and multivariable Cox regression analyses) and the AF group (through univariable Cox regression analysis). In our study, the LAAV threshold for the risk of AF recurrence after ablation (calculated with data from patients in the SR group) was 44 cm/s. This was very close to the 45 cm/s value found in another Polish population treated with cryoballoons [20]. In other studies, the threshold calculated for persistent AF patients ranged between 28 and 37 cm/s [17,18].

The size of the left atrium of the heart undoubtedly influences the results of catheter ablation [21]. When measured using a CT scan, LAVI was shown to be a significant predictor of AF recurrence after cryoballoon ablation [22].

In the current study, the results for the AF group were surprising. We were unable to find any relationship between LASs and the results of AF ablation, even when using univariable analysis. This outcome was in contrast to that of a number of previous publications [23,24]. This disparity was not the result of the study group size, since the sizes of the groups were comparable (90 patients vs. 102 and 94). However, there were some differences in the AF ablation details (e.g., we did not modify the substrate in the first ablation) and in the population (although one of our groups was tested during AF, a substantial number of patients still had paroxysmal AF, while in the mentioned publications, only non-paroxysmal AF patients were considered). Some type of categorization of LASr in future studies may be helpful; however, since there is currently no widely accepted threshold for LASr, we decided not to categorize this variable. Future studies should recalculate the value of LASr in patients tested during AF to determine if this non-invasive test could be helpful in selecting candidates for catheter ablation.

Our analysis had some limitations, including that it was a single-center analysis. After division into groups based on the patient’s cardiac rhythm at the time of echocardiography, the groups became smaller (approximately 100 patients each). Additionally, given that the follow-up was based on clinical information and two 7-day Holter monitoring sessions, some recurrences were possibly missed. Furthermore, because the median follow-up period was 12 months, the data could not be generalized to longer follow-up periods. Our AF recurrence rate was majorly without antiarrhythmic drugs. It is likely that the outcome would have been better if patients had regularly used antiarrhythmic drugs [25]. On the other hand, we used very stringent criteria of AF recurrence (30 s of any atrial tachyarrhythmia), and so, some of the recurrences were possibly not clinically relevant.

## 5. Conclusions

In conclusion, LAAV was found to be an important factor in predicting AF recurrence after AF ablation in patients tested during normal SR, and LAVI was found to be predictive in patients tested during AF. LASr predicted AF recurrence only in univariable analysis in the patients tested during normal SR and had no predictive value in the patients tested during AF.

## Figures and Tables

**Table 1:** Characteristics of the study group. BMI—body mass index, eGFR—estimated glomerular filtration rate, LAAV—left atrial appendage emptying velocity, LAScd—left atrial conduit strain, LASct—left atrial contraction strain, LAEF—left atrial emptying fraction, LASr—left atrial reservoir strain, LAVI—left atrial volume index, LVEF—left ventricular ejection fraction, PA-TDI—total atrial conduction time, SR—sinus rhythm, AF—atrial fibrillaton.

Parameter	Total (*n* = 199)	Patients with SR (*n* = 109)	Patients with AF (*n* = 90)	*p*-Value
Patient characteristics				
Age, years, mean (SD)	62.5 (10.6)	60.7 (11.1)	64.7 (9.5)	0.0076
Woman, *n* (%)	77 (38.7)	50 (45.9)	27 (30.0%)	0.028
BMI, kg/m^2^, mean (SD)	30.1 (4.8)	29.8 (4.7)	30.5 (4.9)	0.30
Non-paroxysmal AF, *n* (%)	75 (37.7)	8 (7.4)	67 (74.4%)	<0.0001
AF duration, years, median (IQR)	3.0 (1.5–5.0)	3.0 (1.5–5.0)	3.0 (1.5–5.0)	0.56
EHRA ^1^, median (IQR)	3 (2–3)	3 (2–3)	2 (2–3)	0.04
Clinical data, *n* (%)				
Hypertension	145 (72.9%)	78 (71.6%)	67 (74.4%)	0.75
Diabetes	41 (20.6%)	21 (19.3%)	20 (22.2%)	0.72
Heart failure	51 (25.6%)	19 (17.4%)	32 (35.6%)	0.005
Vascular disease	42 (21.1%)	20 (18.3%)	22 (24.4%)	0.30
Laboratory data, median (IQR)				
eGFR ^2^, mL/min/1.73 m^2^	90 (90–90)	90 (90–90)	90 (58–90)	0.15
Echocardiographic data, median (IQR)				
LVEF, %	61 (56–64)	63 (60–65)	56 (49–61)	<0.0001
LASr, %	16 (9–26)	25 (19–30)	9.5 (7–12)	<0.0001
LASct, %	−13 (9–16)	−13 (9–16)	(-)	(-)
LAScd, %	−10 (7–14)	−12 (9–15)	−8 (6–11)	<0.0001
LAEF, %	39.5 (27.5–53.5)	52.0 (44.0–59.0)	28.0 (21.0–36.0)	<0.0001
LAVI, mL/m^2^	40.4 (34.1–48.0)	37.0 (31.9–43.4)	45.4 (37.4–58.3)	<0.0001
PA-TDI, ms	156 (144–171)	156 (144–171)	(-)	(-)
E/e’	9 (7–11.5)	8.4 (6.3–10.6)	9.6 (7.7–13.6)	0.006
LAAV (cm/s)	45 (31–63)	58 (42.5–75)	33 (24–44)	<0.0001
Ablation procedure parameters				
AF ablation procedure time, min, mean (SD)	153.3 (39.5)	149.5 (38.7)	157.9 (40.2)	0.14
AF ablation RF time, sec, mean (SD)	1696 (505)	1634 (480)	1771 (527)	0.056
Left atrial pressure max, mmHg, median (IQR)	21 (16–27)	20 (16–26)	22 (17.5–30)	0.0074

^1^ European Heart Rhythm Association AF symptom score. ^2^ eGFR assessed by the local laboratory using the MDRD formula.

**Table 2:** Uni- and multivariable Cox regression analysis for the sinus rhythm patient group (for the meanings of the abbreviations, see Table 1).

	Univariable Analysis			Multivariable Analysis		
	*p*-Value	Hazard Ratio (HR)	HR 95% CI	*p*-Value	Hazard Ratio (HR)	HR 95% CI
Age	0.07	1.034	0.997–1.072			
Sex	0.57	1.215	0.620–2.380			
BMI	0.045	1.078	1.002–1.160	0.06	1.085	0.995–1.182
AF duration (years)	0.96	0.997	0.902–1.013			
Hypertension	0.15	1.822	0.789–4.204			
Diabetes	0.51	1.291	0.602–2.770			
Previous stroke	0.79	0.770	0.105–5.643			
Heart failure	0.14	1.773	0.827–3.802			
Previous myocardial infarction	0.35	1.641	0.575–4.684			
eGFR ^1^ (mL/min/1.73 m^2^)	0.002	0.977	0.962–0.991	0.25	0.989	0.970–1.008
LVEF (%)	0.71	0.991	0.949–1.036			
PA-TDI	0.09	1.016	0.997–1.036			
LASr (%)	0.02	0.953	0.915–0.993	0.26	1.053	0.962–1.154
LASct (%)	0.071	0.945	0.889–1.005			
LAScd (%)	0.04	0.919	0.847–0.997	0.41	0.946	0.826–1.082
LAVI (mL/m^2^)	0.036	1.030	1.002–1.059	0.97	1.001	0.959–1.044
LAEF (%)	0.051	0.974	0.949–1.000			
E/e’	0.007	1.054	1.014–1.095	0.92	1.004	0.931–1.082
AF ablation procedure time (min)	0.74	1.001	0.993–1.010			
AF ablation RF time (s)	0.60	1.000	0.999–1.000			
Left atrial pressure (max., mmHg)	0.001	1.059	1.023–1.095	0.42	1.024	0.962–1.091
LAAV (cm/s)	0.002	0.972	0.955–0.990	0.035	0.974	0.951–0.998
Age	0.07	1.034	0.997–1.072			

^1^ eGFR assessed by the local laboratory using the MDRD formula.

**Table 3:** Uni- and multivariable Cox regression analysis for the AF patients (for the meanings of the abbreviations, see Table 1).

	Univariable Analysis			Multivariable Analysis		
	*p*-Value	Hazard Ratio (HR)	HR 95% CI	*p*-Value	Hazard Ratio (HR)	HR 95% CI
Age	0.97	1.000	0.967–1.036			
Sex	0.95	1.023	0.521–2.010			
BMI	0.35	1.033	0.965–1.105			
AF duration (years)	0.08	1.055	0.993–1.121			
Non-paroxysmal AF	0.024	2.966	1.146–7.674	0.69	1.263	0.408–3.904
Hypertension	0.92	0.964	0.464–2.003			
Diabetes	0.60	1.237	0.561–2.726			
Previous stroke	0.84	0.883	0.270–2.886			
Heart failure	0.84	0.928	0.457–1.883			
Previous myocardial infarction	0.98	1.013	0.309–3.330			
eGFR ^1^ (mL/min/1.73 m^2^)	0.63	0.996	0.979–1.013			
LVEF (%)	0.93	0.998	0.964–1.034			
LASr (%)	0.73	0.983	0.888–1.087			
LAScd (%)	0.08	0.924	0.847–1.008			
LAVI (mL/m^2^)	<0.001	1.040	1.020–1.061	0.03	1.029	1.003–1.056
LAEF (%)	0.28	0.980	0.945–1.016			
E/e’	0.06	1.069	0.995–1.034			
AF ablation procedure time (min)	0.94	1.000	0.993–1.008			
AF ablation RF time (s)	0.78	1.000	0.999–1.013			
Left atrial pressure (max., mmHg)	0.002	1.047	1.016–1.079	0.40	1.020	0.975–1.066
LAAV (cm/s)	0.01	0.964	0.938–0.992	0.71	0.992	0.954–1.032

^1^ eGFR assessed by the local laboratory using the MDRD formula.

## Data Availability

The data that support the findings of this study are available on request from the corresponding author.

## Supplementary Materials

The following supporting information can be downloaded at: https://www.mdpi.com/article/10.3390/jcm12124034/s1, Figure S1: ROC statistics for left atrial appendage emptying velocity (LAAV, sinus rhythm patients only). AUC = 0.69; Figure S2: ROC statistics for left atrial reservoir strain (LASr, sinus rhythm patients only). AUC = 0.62.
