# Supplementary material for: Value of Left Atrial Strain in Predicting Recurrence after Atrial Fibrillation Ablation
Source: J Clin Med. 2023 Jun 13;12(12):4034. doi: 10.3390/jcm12124034 (PMC10299493; doi:10.3390/jcm12124034)
Supplement: Supplementary file 1 [file jcm-12-04034-s001.zip › jcm-2376588-supplementary.pdf]

Supplementary material

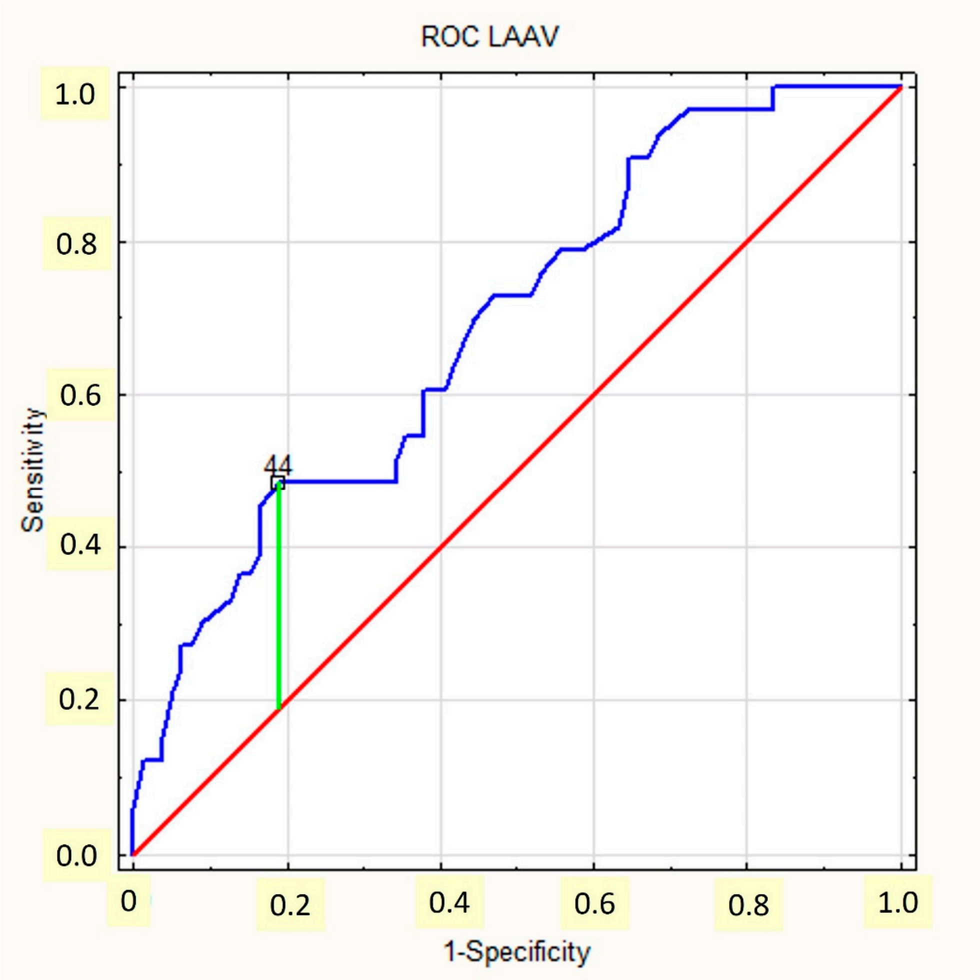

Figure S1. ROC statistics for left atrial appendage emptying velocity (LAAV, sinus rhythm patients only). AUC=0.69.

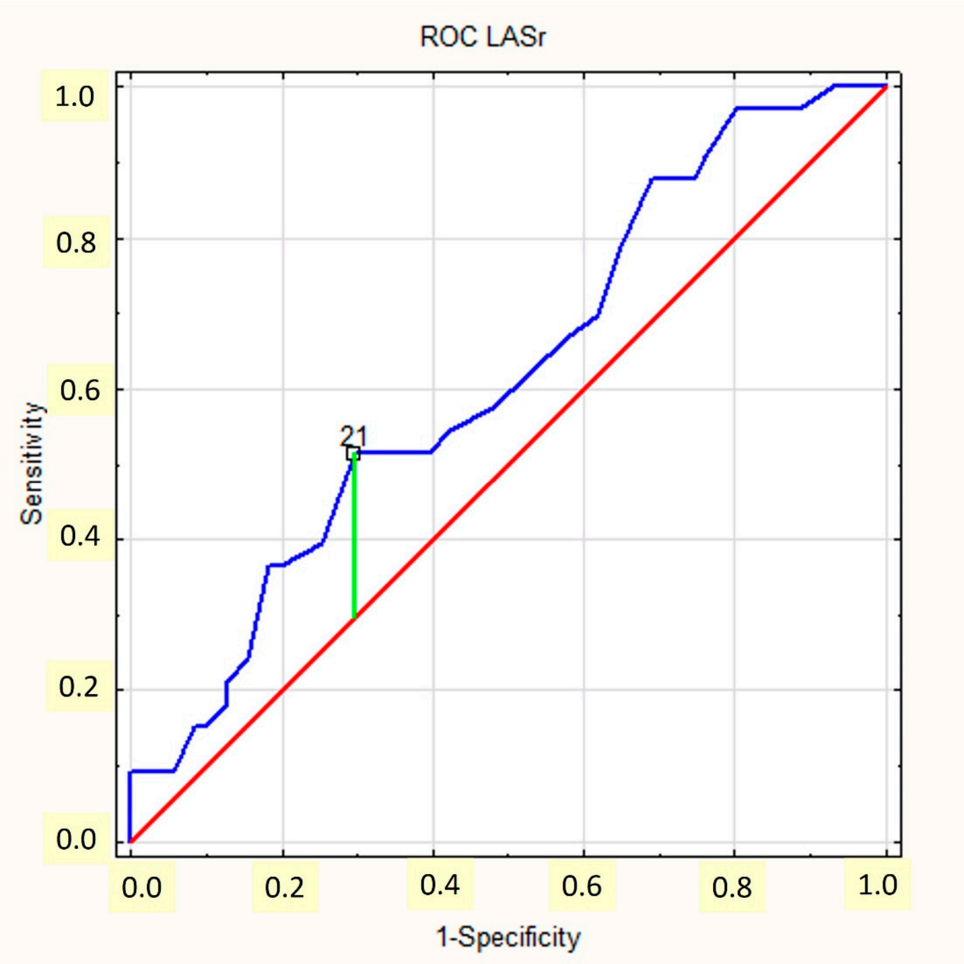

Figure S2. ROC statistics for left atrial reservoir strain (LASr, sinus rhythm patients only). AUC=0.62.
